# DIFFERENTIAL MICRORNAS PROFILING OF MARSHALLESE IN ARKANSAS IS ASSOCIATED WITH CHRONIC DISEASES OF AGING

**DOI:** 10.1093/geroni/igae098.3653

**Published:** 2024-12-31

**Authors:** Gohar Azhar, Ambika Verma, Pankaj Patyal, Sheldon Riklon, Philmar M Kabua, Pearl McElfish, Jeanne Y Wei

**Affiliations:** University of Arkansas for Medical Sciences, Little Rock, Arkansas, United States; University of Arkansas for Medical Sciences, Little Rock, Arkansas, United States; University of Arkansas for Medical Sciences, Little Rock, Arkansas, United States; UAMS Northwest Regional Campus, UAMS Northwest Campus, Arkansas, United States; UAMS Northwest Regional Campus, Fayetteville, Arkansas, United States; UAMS Northwest Regional Campus, Fayetteville, Arkansas, United States; University of Arkansas for Medical Sciences, Little Rock, Arkansas, United States

## Abstract

Arkansas is home to one of the largest populations from the Republic of the Marshall Islands (RMI), who have settled in the United States. Marshallese communities have a disproportionately high prevalence of many chronic diseases including diabetes, obesity, cardiovascular disease and inflammatory conditions. In the era of RNomics, miRNAs are critical in determining the cellular fate in many pathological conditions. Here we report for the first time, miRNAs expression profiling and differential expression in the Marshallese population. Blood samples were collected from 47 Marshallese subjects after obtaining informed consent and subjected to Illumina based next generation RNA sequencing. According to the sequencing reads annotation in the miRBase database, the most significantly expressed miRNAs were screened using log2 fold-change values selected with Bonferroni corrected p-value< 0.01. A total of 63 human miRNAs were differentially expressed in Marshallese subjects, and among them, a total of 52 human miRNAs were significantly upregulated and 11 were significantly downregulated in males versus female subjects. Two most abundant human miRNAs families were significantly upregulated in Marshallese population including hsa-miR-548 and hsa-let-7, which play important role in regulating several inflammatory responses. Interestingly, the differentially expressed miRNAs have known associations with diabetes mellitus (hsa-miR-21, hsa-miR-26, hsa-miR-93, hsa-miR-96, hsa-miR-126), hypertension (hsa-miR-17, hsa-miR-21), Alzheimer’s disease and related dementias (AD/ADRD) (hsa-miR-144-3p, hsa-miR-144-5p) and also cardiovascular diseases (hsa-miR-548j-5p, hsa-miR-584-3p, has-let-7a-5p). Diabetes mellitus remains a major health concern in Marshallese and might accelerate co-morbidities with cardiovascular conditions and ADRD. Hence, the role of specific miRNAs associated with these conditions merits further investigation.

